# Knowledge and Perception of Anti-obesity Medications Among Primary Healthcare Center Visitors in Jeddah, Saudi Arabia in 2024: An Analytical Cross-Sectional Study

**DOI:** 10.7759/cureus.68240

**Published:** 2024-08-30

**Authors:** Renad M Khalaf, Hani A Alghamdi

**Affiliations:** 1 Preventive Medicine, Ministry of Health, Jeddah, SAU; 2 Preventive Medicine, Public Health Directorate, Ministry of Health, Jeddah, SAU

**Keywords:** weight management, pharmacological interventions, obesity treatment, medication awareness, health education, community health

## Abstract

Background: Obesity is a significant public health concern globally, with an alarming prevalence in Saudi Arabia. While anti-obesity medications (AOMs) offer a pharmacological approach to weight management, their awareness and perception remain underexplored.

Objective: To assess the knowledge, perception, and prevalence of AOM usage among primary healthcare visitors in Jeddah, Saudi Arabia.

Methods: A cross-sectional study was conducted among 361 primary healthcare visitors aged 18 to 60 in Jeddah. Data on demographics, knowledge, and usage of AOMs were collected using a structured online questionnaire. The study employed a multistage sampling technique to ensure a representative sample. Statistical analyses included median and interquartile ranges (IQR) for continuous variables and frequencies and proportions for categorical variables. Knowledge differences were assessed using the Mann-Whitney U test and the Kruskal-Wallis test. A pilot study with 10% of the sample was conducted to refine the questionnaire. Approval from the Ethics Committee was obtained, and participants provided informed consent.

Results: The study found a median knowledge score of 44.4% regarding AOMs. Significant associations were noted between higher knowledge levels and educational attainment (p=0.008), healthcare worker status (p=0.009), monthly income (p=0.029), and previous use of weight loss medications (p<0.001). The most commonly used AOM was Ozempic (13.0%). Social media emerged as the most common source of information, positively correlating with higher knowledge levels (p=0.006). Additionally, 72.0% of participants were aware that not everyone is suitable for AOMs, and 78.9% understood that these medications are more effective when combined with diet and exercise. Side effects awareness varied, with 32.1% recognizing nausea and vomiting and 55.4% aware of the increased risks of pancreatitis.

Conclusion: The study reveals that primary healthcare visitors in Jeddah have a moderate level of knowledge about AOMs, with significant differences based on education, professional status, and sources of information. To improve awareness and appropriate use of AOMs, targeted educational interventions should be implemented. These interventions could help reduce obesity-related health issues by ensuring that individuals are well-informed about the benefits and risks of AOMs.

## Introduction

Obesity is a significant public health threat that has seen a threefold increase between 1975 and 2016, evolving into a worldwide epidemic [1]. This condition is characterized by the abnormal or excessive accumulation of fat in the body due to an imbalance between energy intake and expenditure, commonly assessed using a body mass index (BMI) of ≥30 kg/m² [2,3]. However, BMI is often criticized as it fails to distinguish between lean body mass and fat mass [4], suggesting that waist circumference (WC) might be a more reliable measure of visceral and total body fat [5]. Obesity is multifactorial, influenced by dietary habits, sleep patterns, physical activity, medications, and genetic and environmental factors [6,7]. It is associated with increased morbidity, mortality, and high healthcare costs, contributing to over 200 chronic diseases, including hypertension, cardiovascular diseases, type 2 diabetes mellitus, depression, and some malignancies [7]. Consequently, obesity necessitates a multidisciplinary approach and may require lifelong treatment [3].

According to the 2016 World Health Organization report, obesity affected nearly 13% of the global adult population, with a prevalence of about 35.6% in Saudi Arabia [8,9]. A 2013 national-level study highlighted this trend, showing an obesity prevalence of 28.7% [10]. Jeddah, the largest city in the western region of Saudi Arabia, exhibited an alarming obesity prevalence of approximately 35% [11,12]. The treatment of weight-related complications costs Saudi Arabia $3.8 billion, or 4.3% of health expenditure [13]. In response, the Saudi government has implemented the 'Vision 2030' plan, which includes policies aimed at addressing the obesity epidemic to promote a healthier population [9].

Interestingly, studies have shown that over 50% of community pharmacists have inadequate knowledge about the side effects of weight control products, negatively affecting public awareness [14]. Less than one-third of individuals can identify anti-obesity medications (AOMs) as treatments for weight management [15]. This low awareness and negative perception extend to the general population, where most people believe diet and exercise are more effective and safer than AOMs [15]. Various factors, including knowledge, education level, medication adherence, and perception of personal vulnerability, influence the willingness to use AOMs [16-18].

Given the high prevalence of obesity in Saudi Arabia, particularly in Jeddah, this study aims to fill that gap in the limited literature on awareness and perception of AOMs. Understanding the knowledge, perception, and prevalence of AOM usage among visitors to primary healthcare centers (PHCs) in Jeddah in 2024 is crucial. By identifying these factors, targeted interventions can be designed to reduce obesity-related health issues and promote long-term well-being. Previous research has shown that approximately 21% of females in Saudi Arabia use AOMs to lose weight [19]. Therefore, the primary objective of this study is to assess the knowledge, perception, and prevalence of AOM usage among visitors of PHCs in Jeddah, in 2024.

## Materials and methods

Study design and population

The study employed a cross-sectional design targeting primary healthcare visitors in Jeddah, Saudi Arabia. A representative sample of individuals aged 18 and above was included in the study. All individuals residing in Jeddah of both genders and all nationalities were included. Non-Arabic-speaking individuals, those under 18 years old, and patients over 55 years old with pre-existing cardiovascular diseases were excluded from the study.

Sample size and sampling

A probability random sampling method was used in this study. The sample size was calculated based on a 50% estimated prevalence of good knowledge about AOM usage, a margin of error of 5%, a confidence interval (CI) of 95%, and a study power of 80%. The total required sample size was determined to be 385 participants.

A multistage sampling technique was utilized to ensure a representative sample, including the use of cluster sampling. The PHCs in Jeddah were initially divided into clusters based on their affiliation with five different tertiary hospitals: King Abdulaziz Medical City (KAMC), King Fahad General Hospital (KFGH), East Jeddah General Hospital (EGJH), King Abdulaziz General Hospital (KAGH), and Thaghr General Hospital (TGH). Within each selected cluster, one PHCC was randomly chosen. The selected PHCCs were: Abhur Al-Shamaliyah Health Center, Prince Abdul Majeed Health Center, Al-Marwah Health Center, Al-Rawabi Health Center, and Al-Mahjar Health Center. All eligible individuals within each selected PHC were invited to participate in the study.

Data collection methods

Data were collected using a structured questionnaire (see Appendix A) administered through Google Forms (Google Inc., Mountain View, CA, USA). The questionnaire was adopted from a previous study based on the study's objectives and included questions related to awareness, usage, willingness, and alternative weight loss methods [20]. Additionally, participants' demographic and clinical information were collected. Eligible participants were invited to scan a barcode linked to the online questionnaire. Permission to reproduce the questionnaire was obtained (see Appendix B) before data collection [20]. Participants were informed about the study's nature and objectives to obtain informed consent prior to scanning the barcode.

Pilot study

A pilot study was carried out on 10% of the complete sample, consisting of 38 participants, to evaluate the practicality and accuracy of the questionnaire. Participants in the pilot study were recruited from PHCs that were not randomly chosen for the main study. The responses obtained were utilized to enhance the questionnaire and were not included in the final data analysis. However, no adjustments were made based on the findings of the preliminary study.

Statistical analysis

Data were analyzed using SPSS Statistics software (IBM Corp., Armonk, NY, USA). Continuous variables were assessed for normality using the Kolmogorov-Smirnov test and were found to be skewed. Therefore, the median and interquartile ranges (IQR) were utilized to summarize these continuous variables. For categorical variables, frequencies and proportions were calculated. To evaluate participants' knowledge, a total of 11 items were initially included. Subsequently, two items were removed to enhance the internal consistency of the scoring system, improving Cronbach's alpha from 0.74 to 0.80. This resulted in a final set of nine items, each contributing one point for a correct answer. The total scores were then converted into percentages to facilitate interpretation. Statistical significance in knowledge differences was assessed using the Mann-Whitney U test and the Kruskal-Wallis test. These tests reported the mean rank and p-value, with higher mean ranks indicating greater levels of knowledge. The significance level was set at p < 0.05.

Ethical considerations

Ethical approval was obtained from the institutional review board (IRB) of the Directorate of Health Affairs, Jeddah, affiliated with the Ministry of Health in Saudi Arabia (approval no. A01827). All study participants provided informed consent, and their confidentiality and privacy were protected throughout the study. To ensure consent, a preliminary question was asked at the beginning of the study, and only those who consented were directed to complete the form. Collected data were treated confidentially and accessed only by the principal authors for research purposes. Additionally, the consent statement included the names and contact information of the principal author.

## Results

Of the 388 initial responses, 27 were excluded based on the predefined criteria, leaving a final sample of 361 participants. The age ranged from 18 to 60 years. The median age was 28 years, with an IQR of 22 to 38 years. About two-thirds (61.5%) were males and 38.5% were females. The body mass index (BMI) of the participants ranged from 13.9 to 49.6, with a median BMI of 24.9 and an IQR of 21.6 to 28.8. The overall level of knowledge regarding AOMs was assessed and converted into percentages, with results ranging from 0% to 100% and a median knowledge score of 44.4%.

The demographic characteristics and their association with knowledge levels about AOMs among primary healthcare visitors in Jeddah are presented in Table 1. The study included 361 participants, with 70.9% being Saudi and 29.1% non-Saudi. Marital status was diverse, with 47.9% being single, 44.9% married, 5.8% divorced, and 1.4% widowed. Educational levels varied, with a significant portion holding a bachelor's degree (51.2%). Employment status was almost evenly split, with 46.5% employed and 53.5% unemployed. Healthcare workers constituted 32.1% of the sample. Monthly income was reported in various brackets, with 73.1% earning 5,000 Saudi riyal (SAR) or less, and BMI categories ranged from underweight to obesity class III. Smoking status included 24.4% smokers and 75.6% non-smokers. Additionally, 24.4% had previously used weight-loss medications. 

**Table 1:** Demographic characteristics and their association with knowledge levels about AOMs among PHC visitors in Jeddah AOMs: Anti-obesity medications, PHC: Primary healthcare center

Characteristics		N	%	Mean rank	p-value
Gender	Male	222	61.5%	179.73	0.769
	Female	139	38.5%	183.02	
Nationality	Saudi	256	70.9%	185.64	0.148
	Non-Saudi	105	29.1%	169.68	
Marital status	Single	173	47.9%	179.18	0.081
	Married	162	44.9%	189.52	
	Divorced	21	5.8%	152.14	
	Widow	5	1.4%	89	
Educational level	No official education	3	0.8%	65.17	0.008
	Less than high school	12	3.3%	167.75	
	High school	95	26.3%	159.01	
	Diploma	44	12.2%	165.06	
	Bachelor	185	51.2%	199.87	
	Higher education	22	6.1%	172.16	
Employment status	Employed	168	46.5%	178.8	0.706
	Unemployed	193	53.5%	182.92	
Are you a healthcare worker?	Yes	116	32.1%	201.78	0.009
	No	245	67.9%	171.16	
Monthly income	5,000 or less	264	73.1%	176.63	0.029
	5,001-10,000	6	1.7%	298.75	
	10,001-15,000	46	12.7%	177.4	
	More than 15,000	45	12.5%	194.62	
Body mass index (BMI)	Underweight	25	6.9%	159.2	0.469
	Normal	161	44.6%	172.16	
	Overweight	103	28.5%	193.04	
	Obesity class I	49	13.6%	190.91	
	Obesity class II	18	5.0%	196.97	
	Obesity class III	5	1.4%	172.2	
Smoking	Yes	88	24.4%	185.49	0.640
	No	273	75.6%	179.55	
Have you ever used medication to lose weight?	Yes	88	24.4%	204.45	<0.001
	No	273	75.6%	115.33	

Educational level was significantly associated with knowledge levels (p = 0.008), with participants holding a bachelor's degree having the highest mean rank of 199.87, while those with no official education had the lowest mean rank of 65.17. Healthcare workers had significantly higher knowledge levels (mean rank of 201.78) compared to non-healthcare workers (p = 0.009). Monthly income also showed a significant association (p = 0.029), with those earning between 5,001 and 10,000 SAR having a mean rank of 298.75. Lastly, previous use of weight loss medications was significantly associated with higher knowledge levels (p<0.001), with a mean rank of 204.45 for previous users (Table 1). 

The knowledge assessment of AOMs was assessed using nine questions. A significant majority (72.0%) were aware that not everyone is suitable for AOMs, and 78.9% understood that these medications are more effective when combined with diet and exercise. However, only 40.2% correctly identified the mechanism of AOMs. Awareness of side effects was varied: 32.1% recognized nausea and vomiting, 29.4% identified depressed mood, 33.8% knew about diarrhea and constipation, and 25.2% were aware of dizziness and headache. Additionally, 55.4% and 47.6% of participants were aware of the increased risks of pancreatitis and thyroid cancer, respectively. Detailed results are presented in Table 2.

**Table 2:** Knowledge assessment of AOMs among PHC visitors in Jeddah AOMs: Anti-obesity medications, PHC: Primary healthcare center

Questionnaire	Correct		Wrong	
	N	%	N	%
Do you think that anyone can use medications for weight loss?	260	72.0%	101	28.0%
How do anti-obesity medications reduce weight?	145	40.2%	216	59.8%
Do you believe anti-obesity medications are more effective without diet and exercise?	285	78.9%	76	21.1%
Anti-obesity medication side effects include nausea and vomiting	116	32.1%	245	67.9%
Anti-obesity medication side effects include depressed mood	106	29.4%	255	70.6%
Anti-obesity medication side effects include diarrhea and constipation	122	33.8%	239	66.2%
Anti-obesity medication side effects include dizziness and headache	91	25.2%	270	74.8%
Do anti-obesity medications increase the risk of pancreatitis?	200	55.4%	161	44.6%
Do anti-obesity medications increase the risk of thyroid cancer?	172	47.6%	189	52.4%

Regarding attempts and methods of weight loss among PHC visitors in Jeddah, 73.7% reported having tried to lose weight. Dietary modifications were the most common method, with 57.3% engaging in various diets, and low-calorie diets being the most popular (52.6%). Drinking water as a weight loss strategy was used by 44.6%. Specific diets were less common: 21.3% followed a low-carbohydrate diet, 18.6% adhered to a keto diet, and 8.6% followed a plant-based diet. Physical activity was reported by 65.1% of participants, with walking being the most prevalent form (50.7%). Other forms of exercise included cardio (26.0%), running (18.6%), weight lifting (17.7%), swimming (16.1%), bicycling (9.7%), yoga (8.9%), and boxing (5.8%). Other exercises were reported by 10.8% of participants. These findings highlight the diverse range of weight loss methods attempted, with varying degrees of adherence to dietary modifications and physical activity. Find detailed results regarding weight loss attempts in Table 3.

**Table 3:** Methods of weight loss among PHC visitors in Jeddah PHC: Primary healthcare center

Questionnaire	Yes		No	
	N	%	N	%
Have you ever tried to lose weight?	266	73.7%	95	26.3%
Have you ever consulted a specialist to help you lose weight?	115	31.9%	246	68.1%
Do you diet?	207	57.3%	154	42.7%
Are you on a low-calorie diet?	190	52.6%	171	47.4%
Do you drink enough water?	161	44.6%	200	55.4%
Are you on a low-carbohydrate diet?	77	21.3%	284	78.7%
Are you on a keto diet?	67	18.6%	294	81.4%
Are you on a plant-based diet?	31	8.6%	330	91.4%
Do you exercise?	235	65.1%	126	34.9%
Do you walk regularly?	183	50.7%	178	49.3%
Do you do cardio?	94	26.0%	267	74.0%
Do you run?	67	18.6%	294	81.4%
Do you weight lift?	64	17.7%	297	82.3%
Do you swim?	58	16.1%	303	83.9%
Do you go cycling?	35	9.7%	326	90.3%
Do you do yoga?	32	8.9%	329	91.1%
Do you box?	21	5.8%	340	94.2%
Do you do other exercises?	39	10.8%	322	89.2%

The usage of specific AOMs among PHC visitors in Jeddah revealed varying levels of use. Ozempic was the most commonly used AOM, with 13.0% of participants reporting its use. This was followed by Mounjaro at 6.6%, Saxenda at 6.4%, Orlistat at 4.2%, Didrex at 3.3%, and both Contrav and Wegovy being used by 2.2% of participants. Qysmia had the lowest usage rate at 1.4%. These findings highlight the need for increased education and awareness about the benefits and risks of these medications to enhance their appropriate use in weight management (Table 4).

**Table 4:** Usage of specific AOMs among PHC visitors in Jeddah AOMs: Anti-obesity medications, PHC: Primary healthcare center

AOM	Yes		No	
	N	%	N	%
Ozempic	47	13.0%	314	87.0%
Mounjaro	24	6.6%	337	93.4%
Saxenda	23	6.4%	338	93.6%
Orlistat	15	4.2%	346	95.8%
Didrex	12	3.3%	349	96.7%
Contrav	8	2.2%	353	97.8%
Wegovy	8	2.2%	353	97.8%
Qysmia	5	1.4%	356	98.6%

The common sources of information about AOMs among PHC visitors in Jeddah included social media (61.8%), web browsers (45.2%), family and friends (46.5%), and physicians (33.8%). Significant results in terms of knowledge scores were observed for several sources of information. Participants who relied on social media had significantly higher knowledge levels (mean rank of 192.88, p = 0.006) compared to those who did not (mean rank of 161.8). Similarly, those who obtained information from family and friends also demonstrated higher knowledge levels (mean rank of 194.57, p = 0.020) compared to those who did not (mean rank of 169.19). Detailed results are shown in Table 5.

**Table 5:** Sources of information and their association with knowledge levels about AOMs among PHC visitors in Jeddah AOMs: Anti-obesity medications, PHC: Primary healthcare center

Sources of information on AOMs		N	%	Mean rank	p-value
Social media	Yes	223	61.8%	192.88	0.006
	No	138	38.2%	161.8	
Books and medical journals	Yes	60	16.6%	163.12	0.143
	No	301	83.4%	184.56	
Physician	Yes	122	33.8%	193.79	0.094
	No	239	66.2%	174.47	
Web browsers	Yes	163	45.2%	181.8	0.894
	No	198	54.8%	180.34	
My degree study	Yes	58	16.1%	166.97	0.260
	No	303	83.9%	183.68	
Family and friends	Yes	168	46.5%	194.57	0.020
	No	193	53.5%	169.19	
Gym coach	Yes	52	14.4%	174.12	0.604
	No	309	85.6%	182.16	
Other sources of information	Yes	34	9.4%	143.82	0.028
	No	327	90.6%	184.87	

Participants' perceptions of AOMs were assessed and analyzed in relation to their knowledge levels. When asked if they would use AOM if their doctor recommended it, 50.7% of participants responded "yes" with a mean knowledge rank of 180.1, while 49.3% responded "no" with a mean knowledge rank of 181.92 (p = 0.867). Among those who would not use AOMs, 33% cited side effects or complications as their reason, showing a higher mean knowledge rank of 198.34 compared to those who did not cite this reason (mean rank of 172.48, p = 0.026). Participants who preferred other natural or safe alternatives accounted for 16.6% with a mean knowledge rank of 200.46, whereas the remaining 83.4% had a mean rank of 177.12 (p = 0.111). Regarding the price of medications as a barrier, 13.9% of participants reported this concern with a mean knowledge rank of 164.06, whereas 86.1% did not consider price an issue, having a mean rank of 183.72 (p = 0.213). The detailed results of participants' perceptions are shown in Table 6.

**Table 6:** Participant perceptions regarding AOMs and their association with knowledge among PHC visitors in Jeddah AOMs: Anti-obesity medications, PHC: Primary healthcare center

Variable	Response	N	%	Knowledge mean rank	p-value
If your doctor recommended taking anti-obesity medicine, would you use it?	Yes	183	50.7%	180.1	0.867
	No	178	49.3%	181.92	
If not, why?					
Side effects/complications	Yes	119	33%	198.34	0.026
	No	242	67%	172.48	
Not obese/no need	Yes	76	21.1%	187.22	0.556
	No	285	78.9%	179.34	
Other natural/safe alternatives	Yes	60	16.6%	200.46	0.111
	No	301	83.4%	177.12	
Medications prices	Yes	50	13.9%	164.06	0.213
	No	311	86.1%	183.72	
It may affect mental status	Yes	46	12.7%	172.12	0.534
	No	315	87.3%	182.3	
May cause addiction	Yes	35	9.7%	179.94	0.949
	No	326	90.3%	181.11	
Other reasons	Yes	42	11.6%	170.88	0.512
	No	319	88.4%	182.26	

## Discussion

This study highlights the knowledge, perception, and prevalence of AOM usage among PHC visitors in Jeddah in 2024. The findings indicate a relatively high awareness of the limitations and requirements for AOM usage, with 72% of participants recognizing that AOMs are not suitable for everyone and 78.9% understanding the necessity of combining AOMs with diet and exercise. This awareness is crucial for the effective use of AOMs and aligns with existing literature emphasizing the importance of a comprehensive approach to weight management.

The study's results are inconsistent with earlier research indicating low public knowledge about AOMs. For instance, Elrggal et al. found that over 50% of community pharmacists had inadequate knowledge regarding the side effects of weight control products, except for orlistat [14]. Similarly, only 31% of the study participants (712 individuals) identified AOMs as weight management treatments in the Algarni et al. study [15]. This lack of awareness among healthcare professionals may negatively impact public education and AOM usage, reinforcing the need for improved training and resources.

Several factors may contribute to the observed low uptake of AOMs. First, although not the finding of this study, the low level of awareness and negative perception of AOMs may bias the uptake of such medications. According to a cross-sectional study involving 716 individuals from the general population in Saudi Arabia, participants had a good knowledge level about diet (565, 78.9%) and exercise (621, 86.7%). However, there was a lack of knowledge (222, 31%) and a cautious attitude towards AOMs, as most participants believed diet and exercise are more effective, safe, and convenient [15]. In contrast, another study conducted in Hail, Riyadh, and Al-Ahsaa in Saudi Arabia with 1073 participants indicated that 55.6% of them had a generally good level of awareness regarding AOMs. Nevertheless, most of them believed that AOMs increase the risk of pancreatitis (69.3%) and thyroid cancer (64.5%), leading them to prefer not taking AOMs even if prescribed by their physicians [20]. Second, the rapid evolution of AOMs, with new drugs frequently entering the market, may outpace continuing education efforts. This is supported by a study by Rogers et al., which reports that after the recent media attention for Ozempic, multiple nutrient-stimulated stimulated hormone (NuSH) therapies such as dulaglutide, liraglutide, and tirzepatide have entered the market, and this rapid evolution may outpace healthcare professionals' continuing education efforts [21].

This study has its strengths and limitations. One limitation is the reliance on self-reported data, which may introduce response bias. The pilot study's non-randomized sample limits generalizability and the exclusion of certain demographic variables constrains comprehensiveness. The study also didn't assess the long-term impact of educational interventions on AOM knowledge and attitudes, suggesting a need for future research. Despite these limitations, the study has strengths. A sample size of 361 participants provides sufficient power to detect significant associations. The use of validated statistical methods ensures reliable findings. The pilot study refined the questionnaire, enhancing data accuracy and relevance. The diverse participant pool adds to generalizability. The study's detailed examination of information sources about AOMs provides valuable insights for public health education. Overall, this study offers a thorough analysis of AOM knowledge and perception among primary healthcare visitors in Jeddah, contributing to the literature on weight management and educational interventions in public health.

## Conclusions

Our findings highlight the presence of gaps in the knowledge and perception of AOMs among PHC visitors in Jeddah. While awareness of the limitations and necessary adjunctive measures for AOM use is relatively high, overall knowledge remains limited. Addressing these gaps through targeted education and policy initiatives is essential for effective obesity management in Saudi Arabia. Future research should explore longitudinal studies to better understand the long-term impact of AOM awareness and usage on weight management outcomes. Additionally, qualitative studies could provide deeper insights into the barriers and facilitators of AOM usage from both patient and healthcare provider perspectives. Expanding research to include a more diverse population sample across different regions of Saudi Arabia would also be beneficial.

## Figures and Tables

**Figure 1:**
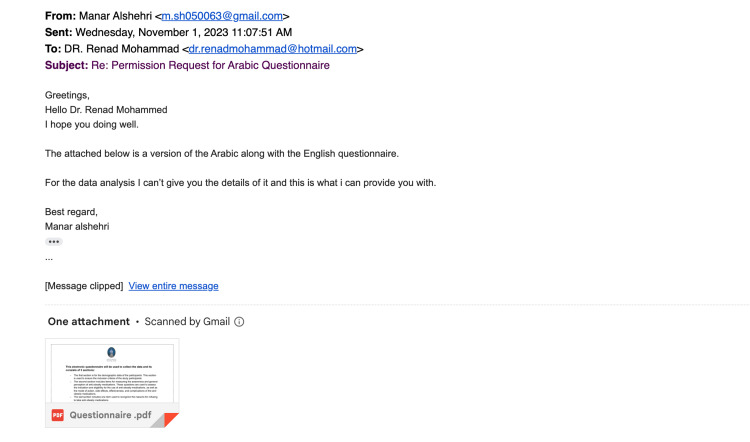
Screenshot of the confirmation email from the corresponding author of the validated questionnaire

## Appendices

Appendix A

A 16-item questionnaire was formed after a thorough literature review. It was translated into Arabic, the native language of the participants. The questionnaire (Tables 7-9) was presented to two consultants to approve its validity.

**Table 7:** The first section of the questionnaire collects the demographic data of the participants This section is used to ensure the inclusion criteria of the study participants.

Demographic characteristics	Response options
1. Gender	Female/Male
2. Age (in years)	Less than 18, 18-39, 40-59, ≥60
3. Residency	Hail, Riyadh, Al-Ahasa
4. Education	High school/Attending university/Bachelor's degree/Higher degree/Others
5. Do you work in a health-related job?	Yes/No
6. Do you have any information about anti-obesity medications?	Yes/No
7. The source of information (if answered 'Yes' to Q6)	Doctor/Relatives/Books/Internet/TV

**Table 8:** The second section of the questionnaire includes items for measuring the awareness and general perception of AOMs These questions are used to assess the indication and eligibility for the use of AOMs, as well as their mode of action, side effects, effectiveness, and complications. AOMs: Anti-obesity medications

Questions	Response options
8. Do you think anyone can use anti-obesity medications to reduce weight?	Yes/No
9. Do you think there are specific BMIs that permit the use of anti-obesity medications?	Yes/No
10. Based on your knowledge, which BMI pindicates the use of these medications in medically free people? (If answered 'Yes' to Q9)	BMI ≥ 25, BMI ≥ 30, BMI ≥ 35, BMI ≥ 40
11. How do you think the anti-obesity medications work?	Reduce the appetite, Enhance the satiety, Decrease the fat absorption, All the above
12. In your opinion, do you think these medications can reduce 3 kg to 8 kg per year?	Yes/No
13. Which of the following do you think is a side effect of the medications?	Nausea, Vomiting, Depressed mood, Diarrhea, Constipation, Dizziness, Headache
14. Based on your knowledge, anti-obesity medications may increase the risk of:	Pancreatitis (Yes/No), Thyroid tumor (Yes/No)
15. Do you think anti-obesity medications are more effective without diet and exercise?	Yes/No

**Table 9:** This third section features one question to determine the reasons for refusing to take AOMs AOMs: Anti-obesity medications

Question	Response options
16. If your doctor recommended taking anti-obesity medicine, would you use it?	Yes/No
17. If 'No', please write why	Free text

Appendix B

Before data collection, permission to reproduce the questionnaire was obtained from the corresponding author [20]. Figure 1 is a screenshot of the email granting permission.
